# Serum ghrelin levels in papillary thyroid carcinoma

**DOI:** 10.1590/2359-3997000000290

**Published:** 2017-09-04

**Authors:** Bekir Ucan, Mustafa Sahin, Muhammed Kizilgul, Mustafa Ozbek, Seyda Ozdemir, Mustafa Calıskan, Erman Cakal

**Affiliations:** 1 SBU Diskapi Yildirim Beyazit Training and Research Hospital Department of Endocrinology and Metabolism Ankara Turkey SBU Diskapi Yildirim Beyazit Training and Research Hospital, Department of Endocrinology and Metabolism, Ankara, Turkey; 2 Ankara University School of Medicine Department of Endocrinology and Metabolism Ankara Turkey Ankara University, School of Medicine, Department of Endocrinology and Metabolism, Ankara, Turkey; 3 SBU Diskapi Yildirim Beyazit Training and Research Hospital Department of Biochemistry Ankara Turkey SBU Diskapi Yildirim Beyazit Training and Research Hospital, Department of Biochemistry, Ankara, Turkey

**Keywords:** Papillary thyroid carcinoma, ghrelin, tumor size

## Abstract

**Objective:**

Ghrelin plays a role in several processes of cancer progression, and numerous cancer types express ghrelin and its receptor. We aimed to investigate serum levels of ghrelin in patients with papillary thyroid carcinoma (PTC) and its association with the prognostic factors in PTC.

**Materials and methods:**

We enrolled 54 patients with thyroid cancer (7 male, 47 female) and 24 healthy controls (6 male, 18 female) in the study. We compared demographic, anthropometric, and biochemical data, and serum ghrelin levels between the groups. Serum ghrelin levels were measured using as enzyme-linked immunosorbent assay.

**Results:**

Ghrelin levels were similar between the groups, but plasma ghrelin levels were significantly higher in tumors larger than 1 cm diameter compared with papillary microcarcinomas. Serum ghrelin levels also correlated with tumor size (r = 0.499; p < 0.001). Body mass index, thyroid-stimulating hormone, and HOMA-IR levels were similar between the groups. There were no statistically significant differences regarding average age and other prognostic parameters including lymph node invasion, capsule invasion, multifocality and surgical border invasion between patients with microcarcinoma and tumors larger than 1 cm.

**Conclusion:**

In our study, no significant difference in serum ghrelin levels was determined between patients with papillary thyroid cancer and healthy controls however, serum ghrelin levels were higher in tumors larger than 1 cm compared to in those with thyroid papillary microcarcinoma.

## INTRODUCTION

Ghrelin, a 28-amino acid peptide hormone, was discovered in 1999 as the endogenous ligand for the growth hormone secretagogue/ghrelin receptor (GHS-R) (1). Ghrelin has well-documented systemic actions including stimulation of gastrointestinal system motility; gastric acid secretion; regulation of sleep, taste sensation, and reward-seeking behavior; modulation of glucose metabolism; suppression of brown fat thermogenesis; regulation of stress and anxiety; prevention of muscle atrophy; and improvement of cardiovascular functions such as vasodilatation and cardiac contractility (2). Ghrelin was implicated in several processes of cancer progression including cell proliferation, cell migration and invasion, angiogenesis, and apoptosis, probably via an autocrine/paracrine mechanism. Pituitary adenomas, gut carcinoids, endocrine pancreatic, ovarian, endometrial, testicular, adrenocortical, prostate, renal, lung, and breast cancer were demonstrated to express ghrelin. Some reports demonstrated that ghrelin may have an inhibitory effect in the proliferation of some cancer types, including thyroid, prostate, and breast cancer, and small cell lung carcinoma (3). Thyroid follicular/parafollicular and thyroid carcinoma cells also express ghrelin (4). The effect of thyroid hormone status on serum ghrelin concentrations was investigated in some studies. Malandrino and cols. observed that plasma ghrelin levels were significantly higher in patients with Hashimoto’s thyroiditis after the levothyroxine treatment maintaining euthyroid state (5). Ruchala and cols. demonstrated ghrelin levels varied depending on hyperthyroid, hypothyroid or euthyroid state in the same patients (6). Biyikli and cols. demonstrated lower ghrelin levels in patients with euthyroid hashimoto thyroiditis (7).

Circulating ghrelin levels were demonstrated as higher in a range of cancer types including colon cancer (8), prostate carcinoma (9), ovarian carcinoma (10), and hepatocellular carcinoma (11) however, no studies have evaluated serum ghrelin levels in papillary thyroid carcinoma. We aimed to investigate serum levels of ghrelin in patients with papillary thyroid carcinoma (PTC) and its association with the prognostic factors in PTC.

## MATERIALS AND METHODS

### Study population

Fifty-four patients (7 male, 47 female) with PTC and 24 age-, sex-, and body mass index (BMI)-matched controls (6 male, 18 female) were included in the study. The mean age of the patients was 42.4 ± 10.1 years and the control group was 42.5 ± 8.9 years. Ethics committee approval and written informed consent of participants were obtained before the study. Histopathologic documents confirmed the diagnosis of PTC. Blood was collected from patients with thyroid cancer before surgery and from healthy individuals as the controls. Subjects with other cancers and autoimmune disorders, hypertension, hepatic or renal dysfunction, diabetes mellitus, or any other inflammatory or medical condition were excluded.

### Clinical, biochemical, and hormonal measurements

Weight, height, waist circumference (WC), hip circumference (HC), and systolic and diastolic blood pressure (BP) were measured. WC was determined by measuring the narrowest point between the costal margin and iliac crest at the end of a normal expiration. BMI was calculated as weight (kg)/height (m)^2^. After an 8-12 hour overnight fast, venipuncture was performed between 8:00 a.m. and 9:00 a.m. and blood samples were collected into plain tubes. Blood samples were centrifuged at 2500 *g* for 15 min within 30 min of collection, and serum samples were stored at –80°C until required for analysis. Serum levels of glucose, insulin, low-density lipoprotein cholesterol (LDL-C), high-density lipoprotein cholesterol (HDL-C), thyroid-stimulating hormone (TSH), free T4 (fT4), antithyroglobulin (Anti-Tg) and antithyroid peroxidase (Anti-TPO) were also measured. The normal range for fT4 was 0.74-1.52 ng/dL. TSH levels ranging between 0.55-4.78 mIU/L was considered normal and normal ranges for anti-Tg and anti-TPO are 0-40 IU/mL and 0-35 IU/mL, respectively.

### Determination of ghrelin in plasma

Blood samples were collected into EDTA-containing tubes and then aprotinin (Phoenix Pharmaceuticals, California, USA) was added immediately. The blood was centrifuged at 1600 x *g* for 15 minutes; after separation of the plasma, it was stored at −80°C until the ghrelin assessment. Measurements of ghrelin were performed in an EPOCH system (BioTek Instruments, Inc, USA) using a commercially available enzyme-linked immunosorbent assay (ELISA) kit (Phoenix Pharmaceuticals, California, USA) following the manufacturers’ instructions. The test range of the ghrelin ELISA kit was 0-100 ng/mL. The specimens were run together in the same experiment.

### Statistical analysis

The descriptive values for the data obtained are expressed as mean ± SD, numbers, and percentage frequencies. Normality was tested using the Kolmogorov-Smirnov test. In addition, the differences between the groups were analyzed using the appropriate Chi-square test. The relationships between individual numeric properties were reviewed using Pearson’s correlation analysis in the patient and control groups. p ≤ 0.05 was used as the level of statistical significance and IBM SPSS 20.0 was used to process the calculations.

## RESULTS

**Figure 1:**
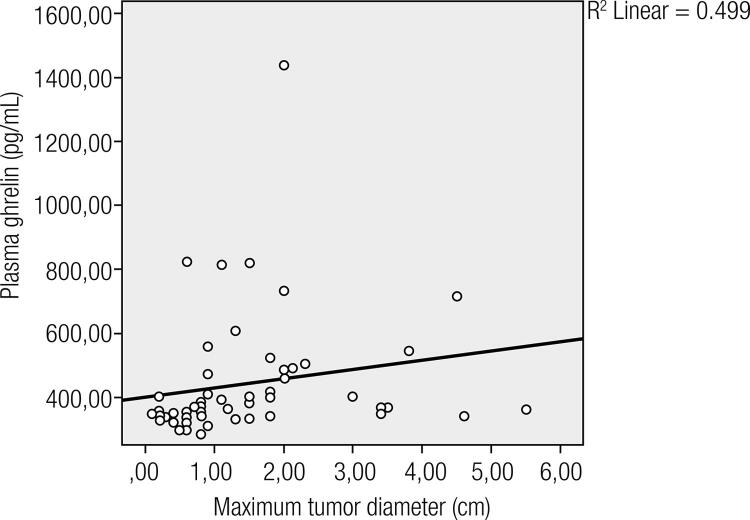
Correlation of tumor size with plasma ghrelin levels.

Fifty-four patients with PTC (mean age: 42.4 ± 10.1 years) and 24 age-, sex-, and BMI matched controls (mean age: 42.5 ± 8.9 years) were enrolled in the study. BMI, WC, TSH levels and insulin resistance (HOMA-IR) were similar between the groups. There was no statistically significant difference in ghrelin levels between patients and controls (p > 0.05) (Table 1). The mean 25-hydroxyvitamin D3 levels were similar between both groups (p > 0.05) (Table 1). Plasma ghrelin levels were significantly higher in tumors larger than 1 cm diameter when compared with papillary microcarcinomas (p = 0.011) (Table 2). There were no statistically significant differences regarding average age and other prognostic parameters including lymph node invasion, capsule invasion, multifocality and surgical border invasion between patients with microcarcinoma and tumors larger than 1 cm (Table 2). Plasma ghrelin levels were correlated with tumor size (r = 0.499; p < 0.001) (Figure 1). Ghrelin levels were not correlated with other parameters including insulin level, fasting plasma glucose, waist circumference, BMI, TSH, 25-hydroxyvitamin D3 level, and age. The proportion of Anti-Tg and Anti-TPO positivity between group with tumor < 1 cm versus group with tumor > 1 cm were similar

**Table 1:** Clinical and laboratory characteristics of patients with thyroid cancer compared with controls

		Controls n = 24	Patients n = 54	P
Age (years)		42.5 ± 9	42.4 ± 10	NS
BMI (kg/m^2^)		28.6 ± 2.5	29.1 ± 5.6	NS
Waist (cm)		96.3 ± 12.1	96.4 ± 12.8	NS
Waist/hip ratio		0.90 ± 0.04	0.91 ± 0.07	NS
Gender				
Male		6	7	NS
Female		18	47	
Ghrelin (ng/mL)		44.1 (26-144)	46.9 (28-141)	NS
Fasting glucose, mg/dL		85 ± 10	87 ± 20	NS
Insulin (mIU/L)		12.7 (5.7-19.7)	11.3 (9.8-12.7)	NS
HOMA-IR (%)		2.96 (0.9-5)	2.5 (2.1-2.9)	NS
Total cholesterol, mg/dL		193 ± 39	207 ± 41	NS
Plasma triglycerides, mg/dL		136 ± 17	145 ± 10	NS
High density lipoprotein cholesterol (mg/dL)		48 ± 10	52 ± 11	NS
Low-density lipoprotein cholesterol (mg/dL)		118 ± 33	120 ± 39	NS
Calcium (mg/dL)		9.6 ± 0.5	9.2 ± 0.7	0.035
Serum phosphorus (mg/dL)		3.4 ± 0.4	3.6 ± 0.8	NS
Vitamin D3 (ng/mL)		16.6 ± 6	14.2 ± 7	NS
TSH (mIU/L)		1.8 (1.4-2.2)	2.3 (1.8-2.8)	NS
Anti-TPO (IU/mL)		104 (22-186)	135 (20-292)	NS
Anti-Tg (IU/mL)		45 (8.5-82)	45 (22.1-67.8)	NS

BMI: body mass index; Anti-Tg: antithyroglobulin; Anti-TPO: antithyroid peroxidase; TSH: thyroid stimulating hormone T4 thyroxine; HOMA-IR: Homeostasis model assessment for insulin resistance.

**Table 2:** Comparison of clinical features and prognostic factors according to tumor size

		Group with tumor size < 1 cm	Group with tumor size > 1 cm	p-value
Age (years)		41.66 ± 10.52	43.07 ± 9.91	0.615
Gender				
F		25	22	0.224
M		2	5	
Central lymph node positivity				
Yes		9	11	0.573
No		18	16	
Capsule invasion				
Yes		3	6	0.273
No		24	21	
Multifocality				
Yes		5	11	0.073
No		22	16	
Lymphadenopathy				
Yes		4	5	0.668
No		23	21	
Surgical border invasion				
Yes		0	2	0.149
No		27	25	
Ghrelin (ng/mL)		374.63 ± 105.66	507.26 ± 237.48	**0.011**
Tumor Size (cm)		0.56 ± 0.26	2.34 ± 1.19	**< 0.0001**
TSH (mIU/L)		1.61 (0.01-9.90)	1.11 (0.02-28.80)	0.572
FT4 (ng/dL)		1.09 (0.82-1.44)	1.27 (0.50-1.97)	0.169
Anti TPO (IU/mL)		39.00 (1.14-1300.00)	33.30 (0.80-1000.00)	0.916
Anti-Tg (IU/mL)		30.00 (10.00-336.00)	20.00 (0.90-500.00)	0.375

## DISCUSSION

Ghrelin was initially isolated from the stomach and demonstrated to robustly stimulate hormone secretion from the anterior pituitary (1). It is well-documented that ghrelin and its receptor, the growth hormone secretagogue receptor (GHSR), are expressed in a broad array of normal tissues and cancer types, and they are considered to function as autocrine/paracrine growth factors. Ghrelin stimulates proliferation in a range of cancer cells, including human hepatoma (12), human erythroleukemia (13), adrenocortical carcinoma (14), pancreatic adenocarcinoma (15), colorectal cancer (16), choriocarcinoma (17), prostate cancer (18), breast cancer (19), and endometrial cancer cell lines (20). Ghrelin regulates apoptosis in cancer. It was shown that ghrelin treatment inhibited apoptosis in endometrial cancer (20), pheochromocytoma (21), and adrenocortical carcinoma cell lines (22). Ghrelin stimulates cell migration and invasion in pancreatic cell lines (15), colorectal cell lines (16), and astrocytoma cells (23).

Nikolopoulos and cols. observed that patients with colon cancer had significantly higher levels of total serum ghrelin. Patients with end-stage disease and patients with poorly differentiated tumors had statistically significantly higher serum total ghrelin levels (8). In contrast to this finding, a study demonstrated that serum ghrelin levels were lower in patients with colorectal carcinoma compared with healthy controls, and patients with end-stage disease had lower ghrelin levels (24). Serum levels of active ghrelin were significantly higher in patients with prostate (18), ovarian (10), and hepatocellular carcinoma (11). Major hyperghrelinemia was observed in end-stage well-differentiated neuroendocrine carcinomas (25). Corbetta and cols. indicated that plasma ghrelin concentrations in patients with gastroenteropancreatic tumors were similar to healthy controls (26). Lin and cols. demonstrated that ghrelin could activate Snail function, thus promoting renal cell carcinoma metastasis, and was associated with unfavorable prognosis (27). Altogether, although there have been a few studies with conflicting results, most studies demonstrated that several cancer tissues expressed and released ghrelin, and it played a role in cancer progression.

Ghrelin expression in the thyroid is related to follicular and parafollicular cells. The association between ghrelin and thyroid cancer has been investigated in a few preclinical and clinical studies. Thyroid carcinomas (medullar, follicular, and papillary) in rats were demonstrated to express ghrelin (28). Ghrelin and GHSR were expressed in human thyroid carcinoma cells (4,29). Volante and cols. also showed that cell proliferation of thyroid carcinoma cells was inhibited by ghrelin (29). Another study demonstrated that although ghrelin alone did not stimulate cell proliferation in thyroid cell lines, it augmented the effects of thyroid stimulating hormone on cell proliferation (30). A recent study demonstrated that ghrelin expression was similar in patients with medullary cancer, papillary cancer, and nodular goiter (31). Karaoglu and cols. indicated that ghrelin tissue levels were lower in papillary carcinoma cells compared with non-cancerous thyroid tissues (32). Morpurgo and cols. reported that ghrelin levels were similar in patients with medullary thyroid cancer and healthy controls (33). We found that ghrelin levels were similar in patients with PTC and controls; however, ghrelin levels were higher in patients with tumor size larger than 1cm compared with papillary microcarcinoma. The prognostic importance of tumor size in PTC is well documented (34).

Endogenous ghrelin stimulates the release of GH, which regulates IGF-1 concentrations (35,36). IGF-1 has mitogenic and antiapoptotic properties (37). Thyroid carcinoma cell lines contain IGF-1 receptors (38). GH might also exert a mitogenic effect by directly inducing c-myc expression (39). In light of this information, we aimed to investigate serum ghrelin levels in papillary thyroid cancer and its association with prognostic factors. We found no difference in ghrelin levels; however, serum ghrelin levels were correlated with tumor size, which is known as an important prognostic factor.

Additionally, decreased vitamin D deficiency and increased HOMA-IR index have been found associated with thyroid cancer (40). Therefore, we evaluated levels of vitamin D3 and the HOMA-IR index. However, we found no relationship between ghrelin and these parameters.

To the best of our knowledge, this is the first study to investigate serum ghrelin levels in patients with PTC. There are some minor limitations to this study such as the relatively small sample size and its single-center design. The presence of thyroiditis could have some influence in ghrelin levels however, it wasn’t analyzed. Additionally, we did not investigate ghrelin receptor expression and its association with plasma ghrelin levels, which would have increased the strength of our study.

In conclusion, no significant difference in serum ghrelin levels was determined between patients with papillary thyroid cancer and healthy controls however, serum ghrelin levels were higher in patients with papillary thyroid cancer with larger tumor size. Further prospective studies investigating ghrelin expression and its association with serum ghrelin levels could be helpful to clarify this issue.
